# Bacterial Communities of the Internal Reproductive and Digestive Tracts of Virgin and Mated *Tuta absoluta*

**DOI:** 10.3390/insects14100779

**Published:** 2023-09-22

**Authors:** Siyan Bi, Xiaodi Wang, Yanhong Tang, Kexin Lei, Jianyang Guo, Nianwan Yang, Fanghao Wan, Zhichuang Lü, Wanxue Liu

**Affiliations:** 1 State Key Laboratory for Biology of Plant Diseases and Insect Pests, Institute of Plant Protection, Chinese Academy of Agricultural Sciences, Beijing 100193, China; 2 Institute of Western Agriculture, Chinese Academy of Agricultural Sciences, Changji 831100, China

**Keywords:** mated, microbial transmission, reproductive tracts, *Tuta absoluta*, 16S rRNA

## Abstract

**Simple Summary:**

*Tuta absoluta* is a worldwide quarantine pest that is highly reproductive and adaptable to the environment, infesting leaves with larvae that can damage a wide range of tomato crops. The microbial community in the insect also changes in response to mating and egg laying. Both reproductive system microorganisms and gut microorganisms can affect the growth and development of the host insects and their ability to mate and reproduce. In this study, we analyzed the bacterial communities in reproductive tissues, gut tissues, and eggs using 16S rRNA, restriction fragment length polymorphism analysis, and QIIME2 dada2 sequencing. Although the diversity of the bacterial communities did not change significantly after male and female mating, this reflects the importance of these genera. In addition, *Enterobacter Kobei* was found in the reproductive organs of males after mating, but its effect on the tomato leafminer needs to be further investigated. Overall, these findings provide a basis for analyzing the potential of tomato leafminer invasion from a microbial perspective and provide new ideas and techniques for designing microbial biocontrol technologies.

**Abstract:**

Microorganisms can affect host reproduction, defense, and immunity through sexual or opportunistic transmission; however, there are few studies on insect reproductive organs and intestinal bacterial communities and their effects on mating. *Tuta absoluta* is a worldwide quarantine pest that seriously threatens the production of Solanaceae crops, and the microbial community within tomato leafminers remains unclear. In this study, 16s rRNA sequencing was used to analyze bacterial communities related to the reproductive organs and intestinal tracts of tomato leafminers (the sample accession numbers are from CNS0856533 to CNS0856577). Different bacterial communities were found in the reproductive organs and intestinal tracts of females and males. Community ecological analysis revealed three potential signs of bacterial sexual transmission: (1) Mating increased the similarity between male and female sex organs and intestinal communities. (2) The bacteria carried by mated individuals were found in unmated individuals of the opposite sex but not in unmated individuals of the same sex. (3) The bacteria carried by unmated individuals were lost after mating. In addition, the abundances of bacterial communities carried by eggs were significantly higher than those of adult worms. Our results confirm that mating leads to the transfer of bacterial communities in the reproductive organs and gut of tomato leafminers, and suggest that this community strongly influences the reproductive process.

## 1. Introduction

There is a close relationship between animals and bacteria, and, through long-term evolution, insects have formed interdependent symbiotic relationships with microorganisms. Symbiotic microorganisms provide nutrients to the host [1], participate in host metabolism [2,3], regulate host behavior and reproduction [4,5,6,7,8], promote growth and development [9], and affect the transmission efficiency of disease vectors [10,11]. The microbiome is a dynamic process that responds to environmental changes such as diet [12,13], climate [14], and time of day [15,16]. Sexual microbiomes come into contact and may interact during mating; however, little is known about the effects of mating on reproduction and gut microbiome composition in invertebrates.

The South American tomato leafminer *Tuta absoluta* (Meyrick) (Lepidoptera: Gelechiidae) has a high biological potential, strong adaptability to various climatic conditions, and rapid colonization ability. As a quarantine pest, it mainly damages Solanaceae crops during larval feeding [17]. The adult insects are mostly active at dusk, and female insects can release female hormones 1–2 days after eclosion to attract male insects for mating. Females can mate only once a day, and can mate six times in their lifetime [18]. They lay approximately 250–300 eggs in a lifetime [19]. The microbial communities in insects also change with egg mating and laying. Microorganisms living in or transmitted through the reproductive tissues of arthropods have a profound effect on the reproduction, health, and evolution of the host. The location and vertical transmission potential of these microorganisms have become key decisive factors in ensuring the adaptability of the host and health of their offspring [20]. In addition to the reproductive organs, the unique structure and physical and chemical properties of the insect gut provide a special environment for a variety of microorganisms. These microorganisms provide important nutrients to the host, assist in food digestion, and improve host defense and detoxification abilities, as well as affect the life span, developmental cycle, and mating and reproduction abilities of the host insect [21,22,23,24,25].

The microorganisms of reproductive tissues are those present in or transmitted through reproductive tissues, and their sources include the environment and other host organisms (horizontal transmission) or movement from parents to offspring (vertical transmission). The location and potential for vertical transmission can be decisive factors in host fitness and offspring health [20]. The surface of an insect egg can serve as a vector for vertical symbiotic transmission from the mother to offspring. Maternally inherited bacteria regulate the host reproductive system in various ways to facilitate vertical transmission. In microbiological studies of brown planthoppers, the taxonomic diversity of female adult fungal communities is usually relatively high. In one study, the abundance of the egg-stage bacterium Acinetobacter was significantly reduced, and the microbial composition of males, females, and male adults differed, which indicated that the microbial community in brown planthoppers was sex-dependent [26]. Reproductive tissue samples of Anopheles mosquitoes have a highly conserved core microbiome containing OTUs of seven bacterial genera with different abundance levels [27]. The bacterial communities in bedbugs reportedly become more similar after the mating of males and females, the sequence variants (SVs) of male and female bedbugs are increasingly efficient, and mating allows new bacteria to be introduced into the mating female and male organs [28]. This suggests an exchange of microorganisms with sex-specific differences during mating. Most ancient obligate bacterial symbionts were transmitted by female insect eggs. Symbiotic bacteria must enter the oocyte of the female insect ovary through follicular cells to spread through the egg. After mating with the female, the male transfers ejaculation through its mating organ (paramere) to the female’s symbiotic mating organ (mesosperm). A few hours later, the sperm passes through the hemolymph into the ovaries [29], where the microorganisms invade other associated tissues, thereby increasing the risk of infection or sperm damage. *Cimex lectularius* studies have demonstrated that mating increases the similarity of the female and male reproductive organs, and bacteria present in mating individuals rather than in virgin individuals are found in the opposite sex; some resident bacteria are replaced by introduction-induced bacteria [28].

Intestinal microorganisms are characterized by rich species, variable niches, extensive feeding habits, and strong adaptability; therefore, they demonstrate a high diversity as well as group diversity at the intraspecific and interspecific levels [30]. The diversity and richness of gut microbes are influenced by several factors, including host species, genotype, diet, and host environment [31,32]. Through long-term evolution, insects and gut microbes have formed interdependent symbiotic relationships. Intestinal microorganisms include archaea, bacteria, fungi, and prokaryotes. Generally, a wide variety of bacteria have substantial advantages, such as *Proteobacteria*, *Firmicutes*, *Actinomycetes*, *Spirochetes*, *Bacteroides*, and *Verrucomicrobia* [33]. Recent studies have reported *Ceratitis capitata* [34], *Bombyx mori* [35], *Apis mellifera* [36], and *Dastarcus helophoroides* [37] as other intestinal microflora. The dominant phyla in the intestinal bacterial community of the fifth- and sixth-instar larvae of the fall armyworm are Firmicutes and Proteus [38]. Antibacterial compounds secreted by *Bacillus subtilis* are the main influencing factor in the reconstruction of the gut microbiota in silkworms [39]. The intestinal microbiota can reflect the physiological state of silkworms, and disturbances in the microbial community can lead to an imbalance in the intestinal homeostasis of silkworms [40]. Microorganisms in the intestinal flora are located outside the cells in the intestinal cavity and are considered endosymbiotic bacteria [41,42], and are essential components of the insect microbiota that affect development and immune functions [43,44]; moreover, they provide necessary nutrients and digestive resources [42]. Bacteria can passed on to the next generation via the egg surface, bacteria-containing capsules, or fecal droplets through vertical transmission and strict coevolution [45,46,47].

We aimed to determine the composition and diversity of the reproductive and intestinal tissues in both sexes of tomato leafminers, the impact of mating on the composition and diversity of the community in the tissues of tomato leafminers, and whether bacterial sexual transfer occurs post-mating.

## 2. Materials and Methods

### 2.1. Insect Breeding

The tomato leafminer population used in the experiment was collected from Yuxi, Yunnan Province. The feeding temperature was 26 ± 2 °C, the relative humidity was approximately 60%, the light cycle was 16L: 8D, and the host plant was *Lycopersicum esculentum* Mill. To facilitate the collection of pupae, the fourth-instar larvae of tomato leafminers were taken in advance, placed in a container with a diameter of 9 cm, and fed fresh tomato leaves. Following pupation, all individuals were divided into sex-specific groups of 20–25 individuals, with a total of five replicates placed in 1.5 mL centrifuge tubes to facilitate eclosion.

### 2.2. Sample Preparation and DNA Extraction

We sequenced the bacterial communities of internal reproductive organs, intestinal tissues, and eggs of the tomato leafminer, including (1) the internal reproductive system of virgin female adults (IRF0), (2) the internal reproductive system of virgin male adults (IRM0), (3) the internal reproductive system of mated females (IRF1), (4) the internal reproductive system of mated males (IRM1), (5) the digestive tract of virgin female adults (DF0), (6) the digestive tract of virgin male adults (DM0), (7) the digestive tract of mated females (DF1), (8) the digestive tract of mated males (DM1), and (9) the eggs (E).

An alcohol lamp was placed next to the dissecting mirror to minimize contamination. Before dissection, the dissection articles were subjected to high-pressure sterilization, and all tweezers were immersed in ethanol (75%) and sterilized with flame.

Adult tomato leafminers reared in the laboratory were dissected and their DNA was extracted (n = 400). Half of the individuals were randomly matched in a 1.5 mL Eppendorf tube. After 1–2 h of successful mating, both were dissected to ensure that the sperm remained inside the mesospermalege of the mated female at the time of dissection. An alcohol lamp was placed next to the dissection microscope to minimize contamination. The dissection kit was autoclaved each day, and all forceps were dipped in ethanol (75%) and flame-sterilized before each dissection.

We collected reproductive and intestinal tissue samples from both sexes (n = 20 per mating status, organ, and sex; see Table 1). The testes and digestive tract were collected from males, and the internal reproductive organs and digestive tract were collected from females. Each tissue was transferred to an Eppendorf tube containing 150 μL of phosphate-buffered saline. All samples were frozen in liquid nitrogen and stored at −80 °C. Each group consisted of five biological replicates.

The dissected samples, including the ovaries, testes, and digestive tracts of the unmatched adults, and the ovary, testes, and intestinal tissues of the mated adults, as well as the collected eggs, were hand-homogenized in an extraction buffer containing 200 μL of lysozyme (50 mg/mL), and then incubated at 37 °C for 1 h to achieve DNA extraction from both Gram-positive and Gram-negative bacteria. DNA was extracted from the samples using the OMEGA Soil Genomic DNA Kit (Omega Bio-Tek, Norcross, GA, USA), following the manufacturer’s instructions. The quantity and quality of DNA were measured using a NanoDrop NC2000 (Thermo Fisher Scientific, Waltham, MA, USA). DNA was stored at −20 °C.

### 2.3. 16S rRNA Gene Amplification and Sequencing

With improvements in sequencing technology, rapid identification and research on microorganisms can be achieved through high-throughput sequencing and other means. In this study, 16S rRNA Restriction Fragment Length Polymorphism (RFLP) analysis and QIIME2 dada2 sequencing technology were used to analyze the bacterial communities in sexual reproductive tissue, intestinal tissue, and eggs.

Based on the concentration, DNA was diluted to 1 ng/μL for use as a PCR template. The V1 region of the 16S rRNA gene was amplified via PCR using the specific primers 27F (5′-AGAGTTTGATCMTGGCTCAG-3′) and 1492R (5′-ACCTTGTTACGACTT-3′), which are effective markers for assessing bacterial diversity [48]. PCR products were mixed in equidensity ratios and purified using a Qiagen Gel Extraction Kit (Qiagen, Germany). Then, the purified product was amplified using PCR, and amplification reactions were conducted in a 25 μL mixture containing 8.25 μL of ddH2O, 5 μL Q5 reaction buffer (5×), 5 μL Q5 high-fidelity GC buffer (5×), 0.25 μL Q5 high-fidelity DNA polymerase (5U/microliter), 2 μL (2.5 mM) dNTPs, 1 μL (10 uM) forward and reverse primers, and a 2 μL DNA template. The PCR cycling conditions were denaturation: 98 °C, 2 min; 25/10 cycles of denaturation (for the first and second amplification steps, respectively): 98 °C, 30 s; annealing: 55 °C, 30 s; extension: 72 °C, 90 s; second extension: 72 °C, 5 min.

### 2.4. 16S rRNA-Based Bacterial Community Analysis

Paired-end reads were assigned to samples based on their unique barcode and truncated by cutting the barcode and primer sequences, which were then merged into raw tags using the DADA2 [49] quality-controlled process. Quality filtering of raw tags was performed under specific filtering conditions to obtain high-quality clean tags using QIIME2 (2019.4). First, the trim-paired primer fragments of the excision sequence were called and the sequences without matching primers were discarded. DADA2 was then invoked with QIIME dada2 denoise-paired for quality control, denoising, concatenation, and removal of chimera sequences.

Sequences with ≥97% similarity were assigned to the same operational taxonomic units (OTUs) using the Vsearch (v2.13.4_linux_x86_64) clustersize module. A representative sequence from each OTU was screened for further annotation. For each representative sequence, the Greengenes database was used based on the QIIME2 classification sklearn (https://github.com/QIIME2/q2-feature-classifier (accessed on 15 October 2021)) algorithm to annotate the taxonomic information. To obtain additional information about the bacterial taxonomic identity, the sequences of all unassigned OTUs were BLASTed against the GenBank database.

The α and β diversity indices were calculated based on the rarefied OTU counts using the QIIME program. The α diversity index represents the diversity in a single sample reflected by parameters, including the richness indices of Chao1 and Observed species, the diversity indices of Simpson and Shannon, Faith’s PD evolutionary diversity index, Pielou’s evenness index, and Good’s coverage index. To evaluate the significance level (*p* < 0.05) among the α diversity of different tissues, an independent sample *t*-test was applied to normally distributed data sets, as determined by the Anderson–Darling test.

Nonparametric tests (Mann–Whitney *U* test) were used for non-normal datasets. The “ggplot2” package in R v3.5.2 was used to display α diversity indices. The β diversity, microbial community variation, was assessed using UniFrac distances within QIIME2. Both weighted and unweighted UniFrac values were calculated and visualized using non-metric multidimensional scaling (NMDS) and principal coordinate analysis (PCoA). NMDS analysis was performed in R v3.5.2 with the “vegan” package. PCoA was displayed using the “ape” package in R v3.5.2. The unweighted Pair-group Method with Arithmetic Means (UPGMA) clustering was performed as a type of hierarchical clustering method to interpret the distance matrix using average linkage and was also conducted in QIIME2. Statistical testing of the variation in microbial community composition was performed using permutational multivariate analysis of variance (PERMANOVA) and analysis of similarity (ANOSIM). The PERMANOVA algorithm is usually labelled with the adonis function or adonis 2 function of the R language “vegan” package, so it is sometimes directly referred to as an adonis analysis. In addition, adonis was performed using the “vegan” package in R v3.5.2 with the default permutation number of 999 (based on Bray–Curtis distances), and the level of significance was set at *p* < 0.05.

## 3. Results

### 3.1. Sequencing Data

We sequenced the communities of five individuals for each organ and mating status, as well as the eggs, and obtained 45 samples. In total, 623,519 sequences were obtained, of which 517,702 were quality filtered (See Table S1 for details), denoised, and dechimerised, resulting in an average of 39 OTUs per sample at a 97% similarity. The sequence length distribution was consistent with the target fragment range, showing that the sequencing analysis was reliable (Figure S1A) and indicating that the sequencing volume was sufficient, the sequencing depth was saturated, and that increasing the sample volume did not produce additional OTUs (Figure S1B). The sample accession numbers are from CNS0856533 to CNS0856577 in https://www.cngb.org/news/detail/691 (accessed on 17 August 2023).

**Figure 1:**
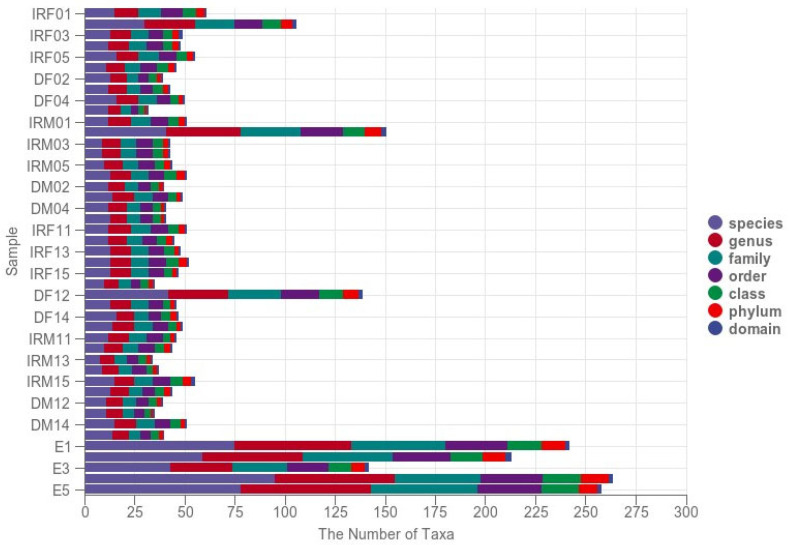
Taxon number statistics of sequenced samples from the internal reproductive system and digestive tract of *T. absoluta*. The vertical coordinate represents each sample, and the horizontal coordinate represents the number of ASV/OTUs (i.e., the number of ASV/OTUs that can only be annotated to these levels) at the highest annotated taxonomic levels of the annotation results for domains, phylums, orders, families, genera, and species, respectively. Different taxonomic levels are identified by different colors, and the height of the columns corresponds to the number of ASV/OTUs. IRF0, internal reproductive system of virgin females; DF0, digestive tract of virgin females; IRM0, internal reproductive system of virgin males; DM0, digestive tract of virgin males; IRF1, internal reproductive system of mated females; DF1, digestive tract of mated females; IRM1, internal reproductive system of mated males; DM1, digestive tract of mated males; and E, eggs within 48 h.

A comparison of the statistical plots of the number of taxonomic units showed that the microbial diversity of eggs was significantly higher (*p* < 0.05) than that of other adult tissues when analyzed at the taxonomic level for domain, phylum, order, family, genus, and species (Figure 1).

### 3.2. Microbiomes of Virgin Tomato Leafminer

**Figure 2:**
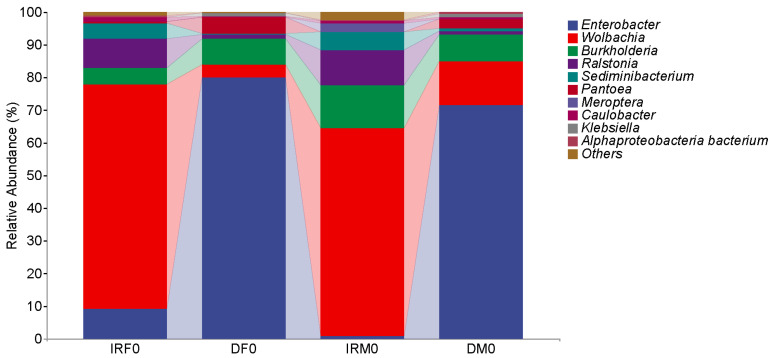
Composition of the top 10 bacteria in relative abundance at the genus level. Each color represents a species, and the height of the color block indicates the proportion of the species in relative abundance. Other species are incorporated as the “Others” shown in the diagram. IRF0, internal reproductive system of virgin females; DF0, digestive tract of virgin females; IRM0, internal reproductive system of virgin males; and DM0, digestive tract of virgin males.

The taxonomic assignment results revealed that the top seven genera (>0.1%) with the 97% sequence identity level in the internal reproductive organs of the virgin female *T. absoluta* were *Wolbachia*, *Enterobacter*, *Ralstonia*, *Burkholderia*, *Caulobacter*, *Afipia*, and *Microbacterium* (Figure 2). Among them, the predominant genus was *Wolbachia*, accounting for 68.73 ± 14.16% of the bacterial sequences in virgin female *T. absoluta*. *Enterobacter* was the subdominant genus, which contributed to 9.63 ± 7.26% of the bacterial sequences in *T. absoluta*. The top six genera (>0.1%) in the gut of the virgin female *T. absoluta* were *Enterobacter*, *Burkholderia*, *Wolbachia*, *Ralstonia*, *Stenotrophomonas*, and *Caulobacter*. Among them, the predominant genus was *Enterobacter*, accounting for 85.97 ± 14.39% of the bacterial sequences in virgin female *T. absoluta*. *Burkholderia* was the subdominant genus, which contributed to 7.82 ± 10.96% of the bacterial sequences in *T. absoluta*. According to the sequencing results, the top four genera of bacteria with a relative abundance in the internal reproductive organs and intestines of virgin females were the same, but the dominant bacteria were different.

The taxonomic assignment results revealed that the top seven genera (>0.1%) with the 97% sequence identity level in the internal reproductive organs of the *T. absoluta* virgin males were *Wolbachia*, *Burkholderia*, *Ralstonia*, *Enterobacter*, *Caulobacter*, *Afipia*, and *Acetobacter*. The predominant genus was *Wolbachia*, accounting for 63.85 ± 17.86% of the bacterial sequences in virgin males of *T. absoluta*. *Burkholderia* was the subdominant genus, which contributed to 13.23 ± 9.11% of the bacterial sequences in *T. absoluta*. The top six genera (>0.1%) in the guts of the virgin male *T. absoluta* were *Enterobacter*, *Wolbachia*, *Burkholderia*, *Ralstonia*, *Caulobacter*, and *Afipia*. Among them, the predominant genus was *Enterobacter*, accounting for 75.65 ± 14.38% of the bacterial sequences in virgin male *T. absoluta*. *Wolbachia* was the subdominant genus, which contributed to 13.35 ± 11.67% of the bacterial sequences in *T. absoluta*. According to the sequencing results, the top six bacterial genera in relative abundance were the same in the internal reproductive organs and intestines of virgin females; however, the dominant bacterial genera were different.

**Figure 3:**
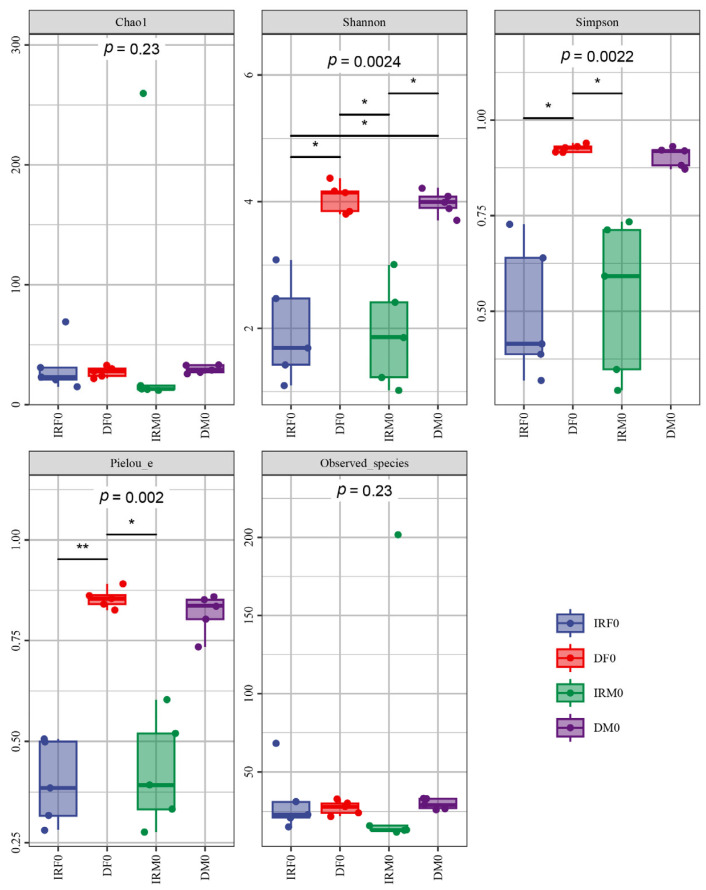
Alpha diversity index analysis. Each panel corresponds to an alpha diversity index, identified in the gray area at the top. In each panel, the horizontal coordinate is the group label and the vertical coordinate is the value of the corresponding alpha diversity index. IRF0, internal reproductive system of virgin females; DF0, digestive tract of virgin females; IRM0, internal reproductive system of virgin males; and DM0, digestive tract of virgin males. *: Significant at the 0.05 level; **: Significant at the 0.01 level, also known as highly significant.

**Figure 4:**
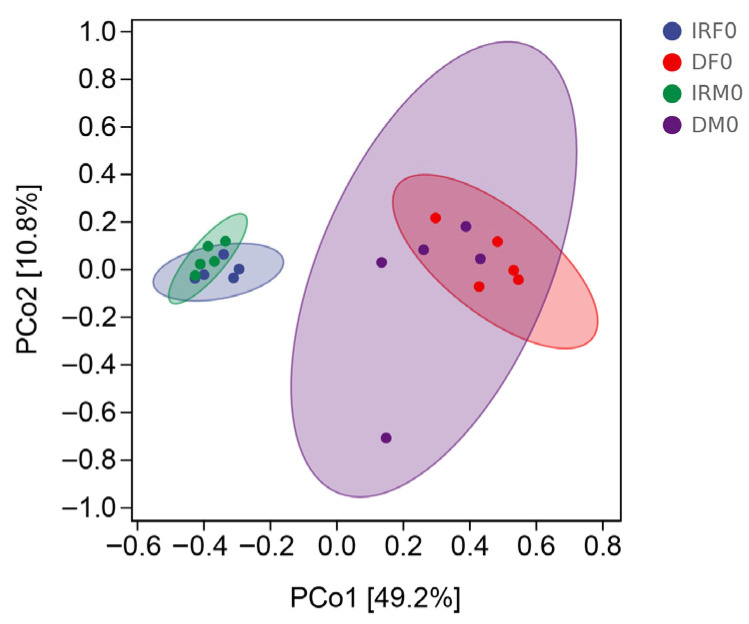
Principal Coordinate Analysis (PCoA) based on Bray–Curtis distance between different sexes and different tissues. IRF0, internal reproductive system of virgin females; DF0, digestive tract of virgin females; IRM0, internal reproductive system of virgin males; and DM0, digestive tract of virgin males.

Our comparative analysis demonstrated that both female and male *Wolbachia* bacteria in the internal reproductive system were the dominant bacteria, whereas *Enterobacter* bacteria were the dominant bacteria in the digestive system. The Chao1 and Observed species richness indices, Shannon and Simpson diversity indices, and Pielou’s evenness of alpha diversity indices showed no significant differences between virgin males and females (*p* > 0.05) (Figure 3). The distance matrix and PCoA analyses (Figure 4) showed that the sample differences between the sexes were not significant.

### 3.3. Mating-Induced Changes in Genital Microbiome

**Figure 5:**
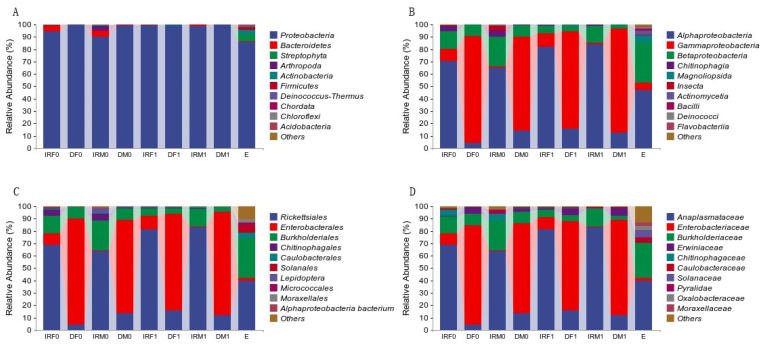
Composition of the top 10 bacteria in terms of relative abundance at the phylum (**A**), class (**B**), order (**C**), and family (**D**) levels. Each color represents a species, and the height of the color block indicates the proportion of the species in relative abundance. Other species are incorporated as the “Others” shown in the diagram. (**A**) Bacterial composition of the internal reproductive system, gut, and eggs of *T. absoluta* at the phylum taxonomic level. (**B**) Bacterial composition of the internal reproductive system, gut, and eggs of *T. absoluta* at the class taxonomic level. (**C**) Bacterial composition of the internal reproductive system, gut, and eggs of *T. absoluta* at the order taxonomic level. (**D**) Bacterial composition of the internal reproductive system, gut, and eggs of *T. absoluta* at the family taxonomic level. IRF0, internal reproductive system of virgin females; DF0, digestive tract of virgin females; IRM0, internal reproductive system of virgin males; DM0, digestive tract of virgin males; IRF1, internal reproductive system of mated females; DF1, digestive tract of mated females; IRM1, internal reproductive system of mated males; DM1, digestive tract of mated males; and E, eggs within 48 h.

At the taxonomic order level, the bacterial 16S rRNA sequences of the reproductive systems, gut tissues, and eggs of both male and female adult tomato leafminers after mating were identified as *Proteobacteria*, *Bacteroidetes*, *Streptophyta*, *Actinobacteria*, *Firmicutes*, and *Deinococcus-Thermus* (Figure 5A). The dominant phylum in all treatments was *Proteobacteria* and the subdominant phylum was *Bacteroidetes*.

At the taxonomic class level, the bacteria in the reproductive system, intestinal tissues, and eggs of both male and female adult tomato leafminers after mating were identified as *Alphaproteobacteria*, *Betaproteobacteria*, *Gammaproteobacteria*, *Chitinophagia*, *Bacilli*, and *Deinococci*. Among them, *Alphaproteobacteria* was the dominant class in the post-mating reproductive system, which was consistent with the pre-mating period: in the post-mating female reproductive system at 82.02 ± 2.34% and the post-mating male reproductive system at 84.25 ± 2.82%. *γ-Amoebacteria* phyla were the dominant phyla in the post-mating gut, consistent with the pre-mating period: in the post-mating female gut at 78.71 ± 3.48% and the post-mating male gut at 84.19 ± 3.51%. The *Betaproteobacteria* content in eggs (33.13 ± 2.67%) was significantly higher than the reproductive system and gut tissues of both male and female adult tomato leafminers before and after mating (Figure 5B).

At the taxonomic order level, the bacteria in the reproductive system, intestinal tissues, and eggs of adult male and female tomato leaf minerals after mating belonged to *Rickettsiales*, *Burkholderiales*, *Enterobacterales*, *Chitinophagales*, *Caulobacterales*, *Micrococcales*, *Rhizobiales*, *Pseudomonadales*, *Bacillales*, *Xanthomonadales*, *Sphingomonadales*, and *Deinococcales*. Among them, *Rickettsiae* were the dominant organisms in the endogamous system, consistent with the pre-mating period: in the female reproductive system after mating at 81.43 ± 2.24% and the male reproductive system after mating at 82.77 ± 3.26%. *Enterobacterales* were the dominant organisms in the intestinal tissues, consistent with the pre-mating period: in female intestines after mating at 78.45 ± 3.51% and male intestines after mating at 83.63 ± 3.52%. the *Rickettsiales* content in eggs was 39.85 ± 7.80%, with 32.99 ± 2.67% of *Burkholderiales*. *Rickettsiales* and *Burkholderiale* were the two most dominant organisms, which was consistent with the pre-mating period (Figure 5C).

At the taxonomic family level, the bacteria in the internal reproductive system, intestinal tissues, and eggs of both male and female adult tomato leafminers belonged to the families *Anaplasmataceae*, *Burkholderiaceae*, *Enterobacteriaceae*, *Chitinophagaceae*, *Caulobacteraceae*, *Oxalobacteraceae*, *Moraxellaceae*, *Bradyrhizobiaceae*, *Xanthomonadaceae*, *Micrococcaceae*, *Intrasporangiaceae*, *Sphingomonadaceae*, *Deinococcaceae*, and *Microbacteriaceae*. *Chitinophagaceae* was the dominant family of the endogamous system, which was consistent with the pre-mating period: in the post-mating female reproductive system at 81.43 ± 2.24% and the post-mating male reproductive system at 82.77 ± 3.26%. *Enterobacteriaceae* was the dominant family of intestinal tissues, which was consistent with the pre-mating period: in post-mating female intestines at 78.45 ± 3.51% and post-mating male intestines 83.63 ± 3.52%. The *Anaplasmataceae* content in eggs was 39.76 ± 7.85%, with 28.68 ± 3.99% of *Burkholderiaceae*. *Anaplasmataceae* and *Burkholderiaceae* were the two most dominant families, which was consistent with pre-mating (Figure 5D).

**Figure 6:**
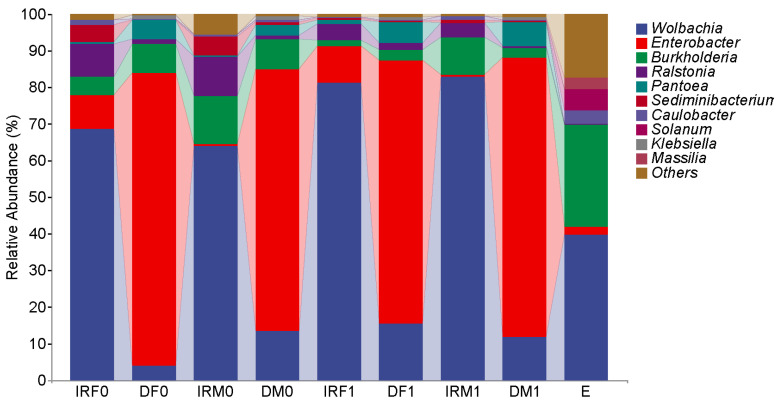
Bacterial composition of the internal reproductive and digestive organs in *T. absoluta* at the genus level. Each color represents a species, and the height of the color block indicates the proportion of the species in relative abundance. Other species are incorporated as the “Others” shown in the diagram. IRF0, internal reproductive system of unmated females; DF0, digestive tract of unmated females; IRM0, internal reproductive system of unmated males; DM0, digestive tract of unmated males; IRF1, internal reproductive system of mated females; DF1, digestive tract of mated females; IRM1, internal reproductive system of mated males; DM1, digestive tract of mated males; and E, eggs within 48 h.

After mating, the dominant bacterial genera in the internal reproductive and digestive organs of male and female insects stayed consistent, and only the relative abundance percentages changed. After mating, *Wolbachia* increased by 12.70% in female internal reproductive organs, 11.55% in female intestines, and 18.93% in male reproductive organs, and slightly decreased by 1.41% in male intestines. The dominant bacterial genus of eggs produced after mating was the same as that of the intra-sexual reproductive system, which was also *Wolbachia*, accounting for 39.76 ± 19.61% of the relative abundance, and the second dominant genus was *Burkholderia*, accounting for 28.12 ± 9.51% of the relative abundance (Figure 6). Analysis of the alpha diversity index showed no significant changes in the Chao1 and Simpson indices before and after mating. No additional bacteria were found in the above studies, but the content of *Burkholderia* are found in the internal reproductive system and digestive tract of male and female adults after mating; in the digestive tract it was the second most dominant bacterium after mating, indicating that the bacterium was transmitted through mating.

To further explore the changes in the bacterial species and richness in insect mating, we further compared the measured sequences in NCBI via Blast, and found that *Enterobacter kobei* did not exist in the internal reproductive organs of unmated males and although it was found in the internal reproductive organs after mating, the content was extremely low (0.15 ± 0.06%, *p* < 0.05). *Enterobacter hormaechei*, *Enterobacter cloacae*, and *Afipia* sp. *BAC308* were only detected in the internal reproductive system of unmated adult females. *Enterobacter hormaechei*, *Enterobacter cloacae*, and *Afipia* sp. *BAC308* were present only in the internal reproductive system of unmated adult females, whereas *alpha proteobacterium PII-14* was only found in the internal reproductive system of adult males after mating. *Microbacterium* sp. *sw0106-31(2)* was present in the internal reproductive systems of unmated female and male adults. Interestingly, this species was not found in the internal reproduction of males after mating; however, the content of *Microbacterium* sp. *sw0106-31(2)* in adult females increased (*p* > 0.05) (Table 2).

During the NCBI comparison and analysis at the species level, *Klebsiella aerogenes* was found in the digestive tract of female adults, as well as in the digestive tract of males after mating. *Enterobacter cloacae* existed only in the digestive tract of female adults before mating, and *Alphaproteobacteria bacterium* existed only in the digestive tract of male adults, signifying it was a unique species (Table 3).

At the species level, the eggs contained some bacteria that are unique to them compared to adults, including *Solanum violaceimarmoratum*, *Janibacter-like* sp. *V4.BO.43*, *Solanum pennellii*, *Caulobacter* sp. *FWC26*, *Asticcacaulis excentricus*, *Arthrobacter* sp. *KAR53*, *Arthrobacter* sp. *JSM 101049*, *Deinococcus petrolearius*, *Staphylococcus warneri*, and *[Empedobacter] haloabium* (Table 4).

**Figure 7:**
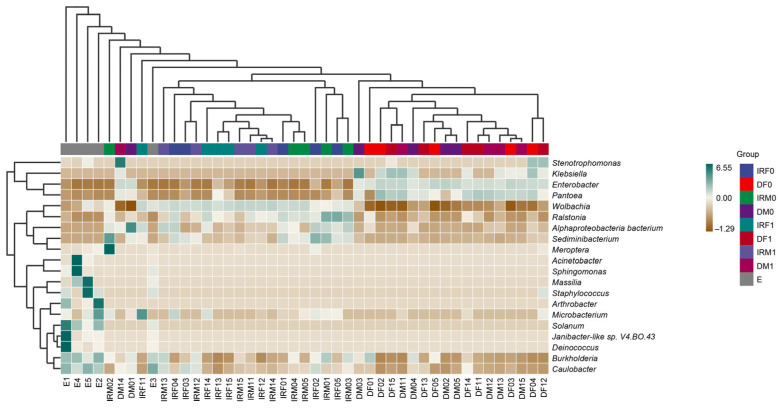
Cluster heat map of the 20 most abundant genera in the bacterial community of *T. absoluta*. The columns represent the samples and the rows represent the bacterial OTUs assigned to the genus level. Dendrograms of the hierarchical cluster analysis grouping genera and samples are shown at the left and top, respectively.

To reveal the dynamics of microorganisms in the adult reproductive system, gut, and eggs, we selected the 20 genera with the highest relative abundances and plotted a relative abundance clustering heatmap (Figure 7). Clustering was based on similarities in species abundance, with horizontal clustering representing sample information and vertical clustering representing species information. The adult reproductive system microorganisms before and after mating as well as the adult gut microbes were similar at the genus level. The main components of microorganisms in the adult reproductive system were *Wolbachia* and *Ralstonia*, and the relative abundance of *Wolbachia* in the adult reproductive system was higher than that in other groups. The main components of adult intestinal microorganisms were *Enterobacter* and *Pantoea*, and the relative abundance of *Enterobacter* in the adult intestinal tract was higher than that in other groups. The relative abundances of *Burkholderia* and *Caulobacter* in egg microorganisms were higher than those in adult tissues.

**Figure 8:**
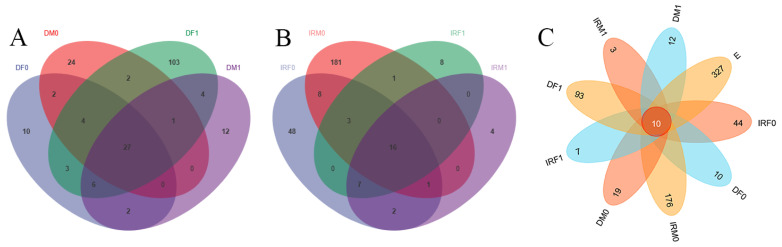
(**A**) Venn diagram of the adult intestinal tract. (**B**) Venn diagram of adult reproductive system. (**C**) All OTUs within the internal reproduction microbiomes split by sex and mating status. Each color block represents a subgroup, the overlapping areas between the blocks indicate the ASVs/OTUs shared between the corresponding groups, and the number in each block indicates the number of ASVs/OTUs contained in that block. IRF0, internal reproductive system of virgin females; DF0, digestive tract of virgin females; IRM0, internal reproductive system of virgin males; DM0, digestive tract of virgin males; IRF1, internal reproductive system of mated females; DF1, digestive tract of mated females; IRM1, internal reproductive system of mated males; DM1, digestive tract of mated males; and E, eggs within 48 h.

To investigate whether species were shared or unique among different samples, Venn diagrams were used for community analysis (Figure 8). Before mating, the female digestive tract contained 54 OTUs and the male digestive tract contained 60 OTUs, of which 33 were shared (Figure 8A). After mating, the female digestive tract contained 150 OTUs and the male digestive tract contained 52 OTUs, of which 38 were shared (Figure 8A). Before mating, the female internal reproductive system had 85 OTUs and the male internal reproductive system had 210 OTUs, of which 28 were shared (Figure 8B). After mating, the female internal reproductive system had 35 OTUs, and the male internal reproductive system had 30 OTUs, of which 23 were shared (Figure 8B). The dataset comprised 701 OTUs (Figure 8C). Ten identical sequences were obtained and their respective microorganisms were identified. Table S2 is based on the 10 ASV sequences mentioned above, and the table of abundance at the sampling level was checked. Five genera were identified in this study: *Wolbachia*, *Burkholderia*, *Enterobacter*, *Ralstonia*, and *Caulobacter*.

**Figure 9:**
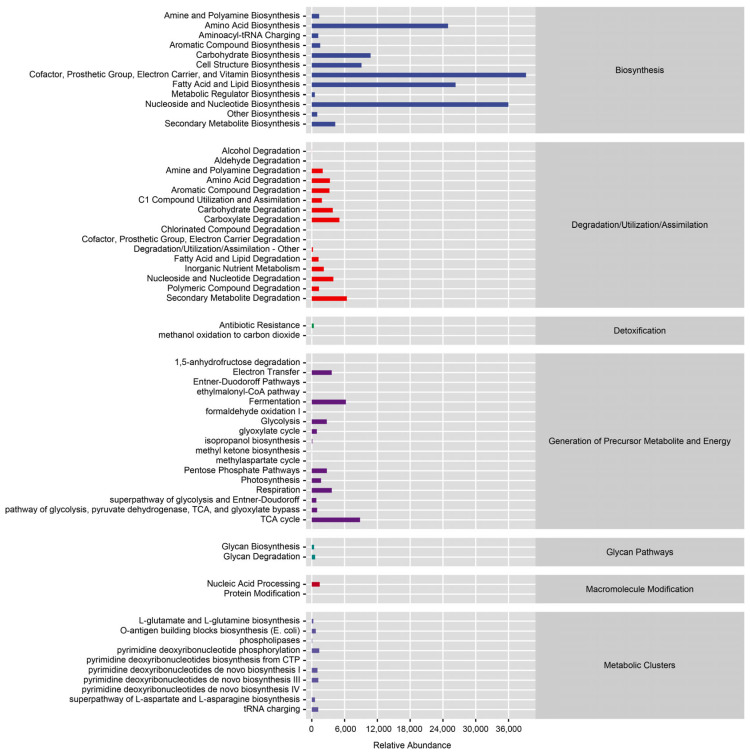
Functional prediction of 16S rRNA microbial metabolic pathways in *T. absoluta*. The horizontal coordinate is the abundance (in units per million KO/PWY/COG) or counts of the functional pathway/classification, the vertical coordinate is the functional pathway/classification at the second level of classification of KEGG/MetaCyc/COG, and the rightmost is the first level of pathway/classification to which the pathway belongs. Shown here is the average abundance of all selected samples or the counts of all selected samples.

To understand the importance of the symbiotic flora of tomato leafminers, Paisano BioPICRUSt2 software was used to predict the functions of bacteria based on 16S rRNA full-length sequencing data. The analysis predicted 417 metabolic pathways, and according to the classification of the METACYC database, the pathways generated by the functional predictions included biosynthesis, degradation/utilization/assimilation, detoxification, generation of precursor metabolites and energy, glycan pathways, macromolecule modification, and metabolic clusters. The top four relatively abundant biosynthesis functions were small molecules (cofactors, prosthetic groups, electron carriers, and vitamins), nucleosides and nucleotides, fatty acids and lipids, and amino acids (Figure 9).

**Figure 10:**
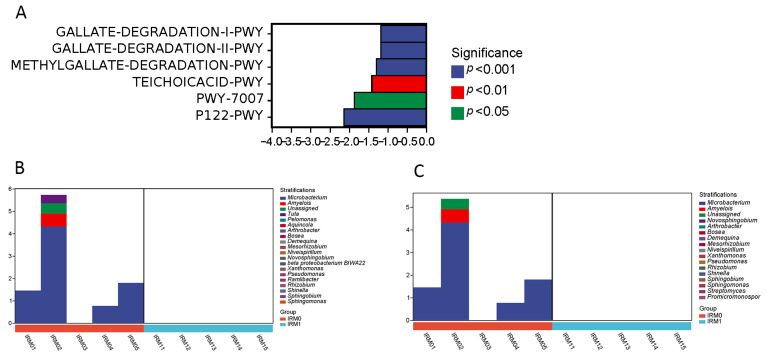
Differential analysis of 16S rRNA microbial metabolic pathways in *T. absoluta* (**A**) and species composition (**B**,**C**). (**A**) Positive values of logFC (log2(fold change)) on the horizontal axis of the graph represent up-regulation of the up-regulated group relative to the control group, and negative values are down-regulation; the vertical coordinates are the different pathway/group labels; the degree of significance is shown in different colors. (**B**,**C**) Horizontal coordinates are sample labels with different colors to identify their grouping attributes; vertical coordinates are the relative abundance of the relevant metabolic pathways; different colors are used to display the proportion of the contribution values of different taxonomic units to the metabolic pathways at the genus level in a hierarchical manner.

After the statistical and differential analysis of the metabolic pathways, the internal reproductive system and digestive tract of male and female individuals were compared before and after mating, respectively, to identify the groups with significant differences. The microorganisms functioning in the GALLATE-DEGRADATION-I-PWY and GALLATE-DEGRADATION-II-PWY pathways were *Microbacterium*, which was not found as a species in the male reproductive system after mating. Comparisons of metabolic pathways in the digestive tract showed no significant differences, with mating having a greater effect on the reproductive system and very little effect on the digestive tract (Figure 10).

## 4. Discussion

The total amount of the symbiotic microorganisms in insects can account for 1–10% of the insect biomass, including vertical transmission (from mother to child), intracellular symbiotic bacteria distributed in special bacteria-containing cells, and extracellular symbiotic bacteria living outside the insect cells (mainly attached to the intestinal parietal cell and free intestinal flora in the intestinal cavity) [50]. In previous studies, *Lactobacillus plantarum* [51], which lives on the surface of intestinal cells, and *Salmonella typhimurium* [52], which lives inside cells, have been identified from mammals.

A previous genome sequencing study demonstrated that the symbiotic microorganisms of most herbivorous insects comprised approximately 30–40 OTU/individuals, among which the symbiotic microorganisms of bees feeding on pollen comprised no more than 6 out/individuals [53]. Studies on *Nilapavata lugens* of the brown planthopper have found at least 18 microbial OTUs [54], and up to 3513 OTU were observed in the *B. tabaci* whitefly [55]. OTU (operational taxonomic unit) is an assumed taxonomic unit, defined as outOTU sequence whose similarity is greater than 97%, to reflect microbial diversity in the sample. In our study, we identified 85 anout10 OTU of the internal reproductive system in unmated females and males, respectively. After mating, thereoutre 35 OTU in the internal reproductive systems of femouts and 30 OTU in the internal reproductive systems of males. A high microbial diversity was observed in the internal reproductive systems and intestinal tracts of tomato leafminers.

Analysis of taxonomic composition showed that, contrary to what was expected from sexual transmission, mating did not cause significant changes in bacterial abundance. This is consistent with the results for *Cimex lectularius* (bedbug). The mating organs of female insects often contain blood cells [29] that can easily devour bacteria as part of insect immune defense [56]. In addition, physical barriers may reduce the uptake of bacteria by females [57].

The microbial community is an important part of the biological environment, containing sexually transmitted microbes (STMs) in the reproductive organs as well as environmental contaminants (or opportunistic microbes, OMs), which are transmitted through the reproductive tract and sexual wounds [58]. This study found that the internal reproductive system of tomato leafminers is mainly composed of *Wolbachia*, *Enterobacter*, *Ralstonia*, and *Burkholderia*. *Wolbachia* species are classified as Gram-negative members of the α-protein bacteria of the *Rickettsiae* order. They affect the evolution, physiology, immunity, and development of the host, and remain relatively stable in the host population [4]. *Wolbachia* is a secondary endosymbiont widely found in a variety of arthropods, and is generally considered a reproductive parasite [4,59], that enhances *Drosophila resistance* to a variety of RNA viruses. In the present study, we also observed that the relative abundance of *Wolbachia* remained the highest after the mating of males and females, and we speculate that this genus was advantageous in the mating process of tomato leafminers. The underlying mechanisms of *Wolbachia*–host interactions have been extensively investigated, and an increasing number of experts in biology have focused on the biological potential of these widespread intracellular bacteria for controlling vector-borne diseases [60]. The key factors that make these bacteria successful are their horizontal transfer across species boundaries and their ability to spread vertically through eggs. *Wolbachia* was also the dominant community bacterium in a diversity analysis of the species composition of eggs after mating [61,62]. This also verified that *Wolbachia* further colonized the host through vertical propagation and continued to affect offspring development.

*Wolbachia*, a symbiotic bacterium found in insects, is a Gram-negative intracellular subsymbiotic bacterium that is widely present in arthropods and is transmitted through eggs. Approximately 65% of insect species naturally carry *Wolbachia*, which is widely distributed in the reproductive tissues of insects, such as eggs, ovaries, and testes. Non-reproductive tissues include the head, muscles, midgut, salivary glands, Martendrian canals, hemolymph, and adipose bodies [63]. *Wolbachia* strongly influences environmental adaptability, reproductive regulation, and viral replication and transmission [64,65]. *Wolbachia* is able to produce individuals that favor transmission by engaging in a variety of mechanisms that regulate host reproductive activity. Its main regulatory mechanisms include cytoplasmic disaffinity, solitary reproduction, feminization, male killing, and enhancement of male or female fecundity [66], and the bacterium promotes the growth and development of its oocytes and normal embryo formation [67,68]. In our study, *Wolbachia* comprised the largest proportion of both the endogenous system and the eggs, so we hypothesize that its function is related to the activities of *T. absoluta* such as mating, egg laying, and the hatching and development of the larvae.

In the present study, *Burkholderia* levels were reduced in the reproductive system of both adults after mating and the bacteria were subdominant in the eggs, thus raising the possibility of vertical transmission of the bacteria. In contrast, the microbial diversity in the eggs was significantly higher than that in adult tissues, suggesting that the presence of these bacteria may facilitate egg hatching. In previous reports, *Burkholderia* has also been found in elongated structures on both sides of the oviduct and within the ovipositor in most species, including *Lagria hirta* [69]. *Burkholderia* has a high potential for biological activity and plays a key role in the defense of the beetle, and by removing the organism experimentally, the eggs become more susceptible to fungal infections, with detrimental consequences for the survival of the larvae [70]. In the present study, *Burkholderia* was widely present in the internal reproductive system and eggs of *T. absoluta*, so it is hypothesized that the fungus also has important functions in *T. absoluta* related to reproduction and larval growth and development.

*Enterobacter kobei* is a new species of the family *Enterobacteriaceae* that resembles *Enterobacter cloacae* and was first used to describe a group of organisms in NHI group 21 [71]. *Enterobacteriaceae* spp. are important opportunistic pathogens. A nursing hospital in Nepal first reported that *E. kobei* was the cause of an outbreak of neonatal sepsis [72]. Currently, little is known about these bacterial species in insects. *Enterobacteriaceae* is considered the main human pathogen in hospital infections, including bacteremia, pneumonia, urinary tract infections, and skin infections [73], and this type of bacteria is often reported with pathogenicity. *Wolbachia*, a widespread secondary endosymbiont of diverse arthropods, is usually considered a reproductive parasite [4,64]. As several studies have failed to show negative effects on host reproductive biology [74,75], researchers have suggested that the high prevalence of *Wolbachia* in such insect species may be explained by the direct fitness benefits conferred on the host by the endosymbiont. Recently, *Wolbachia* conferred enhanced resistance to a variety of RNA viruses in a study on Drosophila [76,77,78].

In previous studies *Diabrotica virgifera* adapted to a corn–soybean rotation by altering the gut microorganism *Klebsiella sp* [79]; in *Bactrocera* dorsalis in vivo *Klebsiella michiganensis* can increase host tolerance to low-temperature stress by stimulating host arginine and proline metabolic pathways [80]; in orange fruit fly *Bactrocera* dorsalis and melon fruit fly *Bactrocera cucurbitae* Klebsiella was found to be able to improve host tolerance to low-temperature stress; compounds produced by *Klebsiella oxytoca* were found to attract females in the orange fruit fly *Bactrocera* dorsalis and the melon fly *Bactrocera* cucurbitae [7,8]; *Klebsiella* spp. are involved in insect growth and immunity [81]. In our study, Klebsiella aerogenes was present in the digestive tract of *T. absoluta*, and it is hypothesized that this bacterium can influence the feeding choice and digestion of *T. absoluta*; due to the presence of this bacterium before and after mating, it is hypothesized that this bacterium may also have a function related to its reproduction.

Insects are colonized by numerous microorganisms. During the process of coevolution, a symbiotic relationship has been established between insects and microorganisms. Insects provide a stable habitat for bacteria and share a specific metabolic pathway, and symbiotic bacteria can assist host nutrition metabolism, provide nutrients, and promote insect growth and reproduction [82]. Bacteria in the foregut of Cassida rubiginosa also contain pectin-digestion genes, and beetles obtain amino acids and vitamins via bacterial decomposition [83]. In addition, *Buchnera aphidicola*, which is incubated with *Acyrthosiphon pisum*, provides essential amino acids to the host [84], and *Buchnera* is maternally transmitted vertically between parents and offspring [85,86]. Thus, endosymbionts may strongly promote insect invasion. Opportunities for horizontal transmission arise when different insect species from different populations or populations of invasive species come into contact with endosymbionts that harbor different symbionts. The acquisition of endosymbiosis from the environment leads to genotype × genotype interactions (host–symbiont recombination) and may lead to ad hoc acquisition of specific new traits, enhanced stress tolerance, and resistance to parasites. This may have enhanced the invasive potential of this species [87]. However, few studies have linked symbiont-mediated traits to invasion potential [88]. In the hybridization of type B in *Bemisia tabaci* with the native biotype, the frequency of *Arsenophonus*-carrying hybrids was significantly lower than expected, indicating a loss of this endosymbiont in viable hybrids or that the viability of the hybrids carrying it was decreased [89].

## 5. Conclusions

The range of tomato leafminers in China is expanding, posing a serious threat to China’s agricultural development. However, at present, our understanding of the bacterial communities in the internal reproductive and digestive systems of tomato leafminers is limited. In this study, 16S rRNA sequencing technology was used to analyze and count bacterial communities in the internal reproductive and intestinal organs of female and male tomato leafminers before and after mating. First, we clarified the composition and qualitative results of the diversity of bacterial communities in the internal reproductive and digestive systems of tomato leafminers as well as the distribution of dominant bacteria. Although the diversity of the bacterial community did not change significantly after male and female mating, it reflected the importance of these genera. In addition, *Enterobacter kobei*, an opportunistic pathogen, was found in the internal reproductive organs of males after mating, but its impact on tomato leafminers requires further study. The above results are helpful for deepening our understanding of the bacterial communities of tomato leafminers and further using the characteristics of the symbiotic community to effectively control them. The use of the microorganisms in tomato leafminers is an alternative biological control strategy. The results of this study show that the reproductive system, intestinal tissues, and egg bacterial communities of adult tomato leafminers are relatively diverse, which can provide new ideas and techniques for analyzing the invasion potential of tomato leafminers from the perspective of symbiotic microorganisms and designing microbial-based biological control techniques.

## Figures and Tables

**Table 1:** Sample information of *T. absoluta* used for amplicon sequencing.

Sample	Organ	Sex	Mating Status	Total Number
IRF0	Internal Reproductive System	Female	Virgin	20 × 5
IRM0	Internal Reproductive System	Male	Virgin	20 × 5
IRF1	Internal Reproductive System	Female	Mated	20 × 5
IRM1	Internal Reproductive System	Male	Mated	20 × 5
DF0	Digestive tract	Female	Virgin	20 × 5
DM0	Digestive tract	Male	Virgin	20 × 5
DF1	Digestive tract	Female	Mated	20 × 5
DM1	Digestive tract	Male	Mated	20 × 5
Egg	-	-	-	200 × 5

**Table 2:** Species-level bacterial community distribution in internal reproductive organs of IRF0 (internal reproductive system of unmated females), DF0 (digestive tract of unmated females), IRM0 (internal reproductive system of unmated males), and DM0 (digestive tract of unmated males).

ID	IRF0	IRM0	IRF1	IRM1
*Wolbachia endosymbiont of Bryobia spec. I VIDR-2008*	68.73 ± 5.66%	67.34 ± 8.03%	81.43 ± 2.24%	82.76 ± 3.25%
*Burkholderia cenocepacia*	5.05 ± 1.12%	14.29 ± 4.40%	1.54 ± 0.39%	10.19 ± 3.12%
*Ralstonia* sp. *1F2*	8.93 ± 2.55%	12.19 ± 2.43%	4.47 ± 1.19%	3.92 ± 0.85%
*Sediminibacterium salmoneum*	4.73 ± 0.91%	3.90 ± 1.11%	0.69 ± 0.15%	0.79 ± 0.31%
*Enterobacter cancerogenus*	2.02 ± 0.80%	0.23 ± 0.19%	4.22 ± 1.25%	0.36 ± 0.16%
*Enterobacter asburiae*	1.40 ± 0.32%	0.08 ± 0.03%	2.50 ± 0.67%	0.13 ± 0.07%
*Enterobacter kobei*	1.04 ± 0.59%	0.00%	2.23 ± 1.18%	0.15 ± 0.06%
*Caulobacter* sp.	1.23 ± 0.37%	0.67 ± 0.05%	0.23 ± 0.07%	1.02 ± 0.32%
*Enterobacter hormaechei*	2.60 ± 1.69%	0.00%	0.00%	0.00%
*Enterobacter cloacae*	1.82 ± 1.15%	0.00%	0.00%	0.00%
*Pantoea* sp. *NJ-32*	0.43 ± 0.25%	0.00%	1.10 ± 0.39%	0.00%
*Alphaproteobacteria bacterium*	0.51 ± 0.14%	0.47 ± 0.16%	0.26 ± 0.10%	0.22 ± 0.13%
*Enterobacter* sp. *CTSP4*	0.32 ± 0.19%	0.00%	0.85 ± 0.45%	0.00%
*Microbacterium* sp. *Sw0106-31(2)*	0.15 ± 0.07%	0.06 ± 0.02%	0.28 ± 0.19%	0.00%
*Afipia* sp. *BAC308*	0.10 ± 0.03%	0.00%	0.00%	0.00%
*alpha proteobacterium PII-14*	0.00%	0.00%	0.00%	0.04 ± 0.02%
*Others*	0.57 ± 0.46%	0.06 ± 0.04%	0.12 ± 0.06%	0.09 ± 0.05%

**Table 3:** Species-level bacterial community distributions in digestive tract of *T. absoluta*.

ID	DF0	DM0	DF1	DM1
*Enterobacter cancerogenus*	32.79 ± 2.24%	21.34 ± 1.09%	30.72 ± 1.95%	33.50 ± 1.89%
*Enterobacter asburiae*	26.93 ± 3.44%	23.94 ± 6.44%	18.67 ± 2.23%	19.43 ± 1.76%
*Enterobacter kobei*	12.61 ± 3.29%	7.82 ± 1.59%	16.18 ± 2.63%	17.40 ± 1.63%
*Wolbachia endosymbiont of Bryobia spec. I VIDR-2008*	4.01 ± 1.34%	13.35 ± 4.67%	14.34 ± 3.11%	11.94 ± 3.36%
*Burkholderia cenocepacia*	7.82 ± 4.39%	8.22 ± 2.75%	3.43 ± 0.94%	2.70 ± 0.87%
*Pantoea* sp. *NJ-32*	5.19 ± 1.55%	2.84 ± 0.48%	6.67 ± 0.74%	6.41 ± 0.61%
*Enterobacter* sp. *CTSP4*	4.83 ± 1.31%	2.59 ± 0.64%	5.20 ± 0.68%	5.16 ± 0.25%
*Ralstonia* sp. *1F2*	1.47 ± 0.84%	1.05 ± 0.43%	2.07 ± 0.50%	0.64 ± 0.29%
*Klebsiella aerogenes*	0.64 ± 0.27%	0.00%	0.82 ± 0.50%	0.97 ± 0.36%
*Enterobacter cloacae*	1.03 ± 0.67%	0.00%	0.00%	0.00%
*Caulobacter* sp.	0.21 ± 0.08%	0.55 ± 0.20%	0.49 ± 0.15%	0.29 ± 0.14%
*Sediminibacterium salmoneum*	0.17 ± 0.11%	0.68 ± 0.36%	0.18 ± 0.04%	0.11 ± 0.03%
*Alphaproteobacteria bacterium*	0.00%	0.46 ± 0.36%	0.00%	0.11 ± 0.05%
*Others*	0.23 ± 0.10%	0.04 ± 0.02%	0.32 ± 0.24%	0.20 ± 0.12%

**Table 4:** Species-level bacterial community distribution in eggs of *T. absoluta*.

ID	E
*Wolbachia endosymbiont of Bryobia spec. I VIDR-2008*	39.76 ± 7.85%
*Burkholderia cenocepacia*	27.86 ± 3.78%
*Solanum violaceimarmoratum*	4.82 ± 1.84%
*Caulobacter* sp.	2.88 ± 0.48%
*Enterobacter asburiae*	1.32 ± 1.01%
*Janibacter-like* sp. *V4.BO.43*	0.93 ± 0.75%
*Solanum pennellii*	0.92 ± 0.27%
*Caulobacter* sp. *FWC26*	0.76 ± 0.48%
*Asticcacaulis excentricus*	0.67 ± 0.32%
*Arthrobacter* sp. *KAR53*	0.66 ± 0.56%
*Arthrobacter* sp. *JSM 101049*	0.56 ± 0.31%
*Deinococcus petrolearius*	0.51 ± 0.42%
*Enterobacter cancerogenus*	0.44 ± 0.11%
*Staphylococcus warneri*	0.40 ± 0.20%
*[Empedobacter] haloabium*	0.37 ± 0.16%
Others	13.03 ± 2.48%

## Data Availability

The 16S rRNA sequences of bacteria data (CNP0004565) that support the findings of this study are openly available in CNSA of CNGBdb at https://db.cngb.org/cnsa/ (Accessed on 22th September 2023).

## Supplementary Materials

The following supporting information can be downloaded at https://www.mdpi.com/article/10.3390/insects14100779/s1, Figure S1: 16S rRNA sequence length distribution (A) and rarefaction curve (B).; Table S1: Basic information of 16S rRNA high-throughput sequencing of *T. absoluta*; Table S2: Basic information of 16S rRNA high-throughput sequencing of *T. absoluta*.
