# Supplementary material for: Bacterial Communities of the Internal Reproductive and Digestive Tracts of Virgin and Mated Tuta absoluta
Source: Insects. 2023 Sep 22;14(10):779. doi: 10.3390/insects14100779 (PMC10606990; doi:10.3390/insects14100779)
Supplement: Supplementary file 1 [file insects-14-00779-s001.zip › insects-2589615-supplementary.pdf]

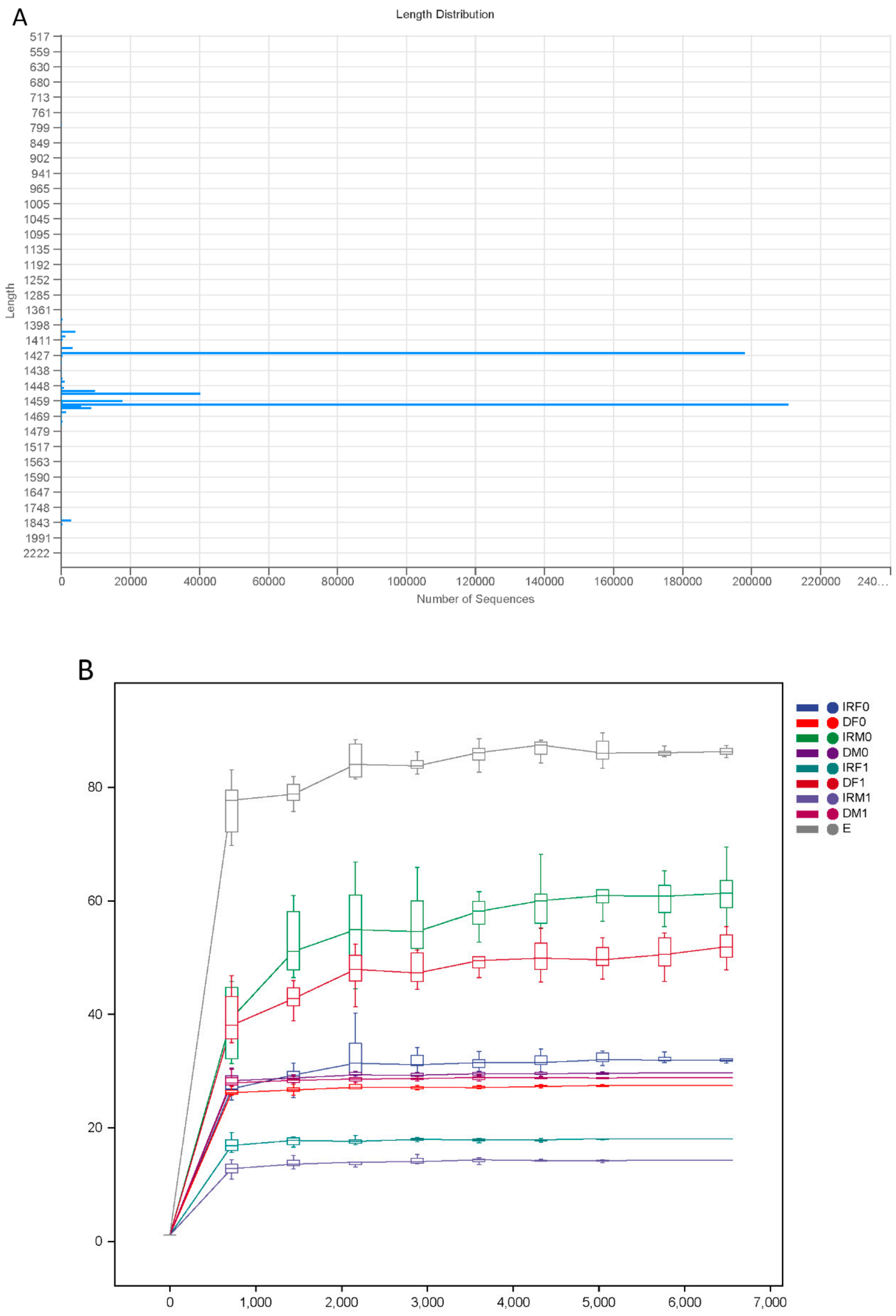

Figure S1: 16S rRNA sequence length distribution (A) and rarefaction curve (B). The 16S rRNA sequence lengths of nine treatment groups of *T. absoluta* were analyzed, and fragments ranged from 1400 bp to 1500 bp (A); As the sequencing depth increased, the dilution curves for all groups of samples decreased in slope as the number of samples sequenced increased, and the curves gradually leveled off, indicating that continuing to increase the number of samples sequenced would only produce a small number of OTUs.

Table S1: Basic information of 16S rRNA high-throughput sequencing of *T. absoluta*

Group	Number of Input	Number of Denoised
IRF0	12344±563	10619±488
IRM0	9426±607	8260±544
IRF1	10779±453	9389±414
IRM1	10862±946	9445±829
DF0	12595±939	10599±790
DM0	11224±947	9485±796
DF1	12106±1148	10194±952
DM1	1288±1611	10496±1245
E	12165±2414	10144±1989

The first column in the table is the grouping of samples; the second column is the amount of sequences of the original data; and the third column is the amount of denoised sequence data.

Table S2: Common microorganisms represented by the Venn diagram in Figure 9

**Sample ID**	**Species represented**
ASV_91	*d__Bacteria; p__Proteobacteria; c__Alphaproteobacteria; o__Rickettsiales; f__Anaplasmataceae; g__Wolbachia; s__unclassified*
ASV_535	*d__Bacteria; p__Proteobacteria; c__Betaproteobacteria; o__Burkholderiales; f__Burkholderiaceae; g__Burkholderia; s__unclassified*
ASV_726	*d__Bacteria; p__Proteobacteria; c__Gammaproteobacteria; o__Enterobacterales; f__Enterobacteriaceae; g__Enterobacter; s__unclassified*
ASV_627	*d__Bacteria; p__Proteobacteria; c__Betaproteobacteria; o__Burkholderiales; f__Burkholderiaceae; g__Ralstonia; s__unclassified*
ASV_376	*d__Bacteria; p__Proteobacteria; c__Gammaproteobacteria; o__Enterobacterales; f__Enterobacteriaceae; g__Enterobacter; s__unclassified*
ASV_45	*d__Bacteria; p__Proteobacteria; c__Betaproteobacteria; o__Burkholderiales; f__Burkholderiaceae; g__Burkholderia; s__unclassified*
ASV_326	*d__Bacteria; p__Proteobacteria; c__Gammaproteobacteria; o__Enterobacterales; f__Enterobacteriaceae; g__Enterobacter; s__unclassified*
ASV_613	*d__Bacteria; p__Proteobacteria; c__Betaproteobacteria; o__Burkholderiales; f__Burkholderiaceae; g__Burkholderia; s__unclassified*
ASV_781	*d__Bacteria; p__Proteobacteria; c__Gammaproteobacteria; o__Enterobacterales; f__Enterobacteriaceae; g__Enterobacter; s__unclassified*
ASV_40	*d__Bacteria; p__Proteobacteria; c__Alphaproteobacteria; o__Caulobacterales; f__Caulobacteraceae; g__Caulobacter; s__unclassified*

The table shows the ASV counterparts of the sequenced samples.
